# The comparison of accuracy and practicability between ultrasound and spiral CT in the diagnosis of intestinal obstruction: A protocol for systematic review and meta-analysis

**DOI:** 10.1097/MD.0000000000023631

**Published:** 2021-01-29

**Authors:** Qingya Yang, Fan Zhao, Junfeng Qi, Long Su

**Affiliations:** Wuwei People's Hospital, Wuwei, Gansu Province, China.

**Keywords:** intestinal obstruction, protocol, spiral computed tomography, systematic review, ultrasound

## Abstract

**Background:**

Acute abdominal pain is often caused by intestinal obstruction, with high morbidity, and mortality, so that the early diagnosis is particularly important. Currently, both spiral CT and ultrasound are common imaging diagnostic methods. However, the accuracy and practicality of the diagnosis are controversial. Therefore, the purpose of this study is to systematically evaluate the accuracy and practicality of spiral CT and ultrasound in the diagnosis of intestinal obstruction.

**Methods:**

Retrieval of English database (PubMed, Embase, Web of Science, the Cochrane Library) and Chinese database (CNKI, WAN FANG, VIP, CBMDISC) by computers. From the establishment of the database to October 2020, a diagnostic experimental study on the diagnosis of intestinal obstruction by ultrasound and spiral CT was conducted. Two researchers independently conducted data extraction and quality evaluation of literature on the included studies, and Meta Disc1.4 and RevMan5.3 were used for meta-analysis on the included literature.

**Results:**

Sensitivity, specificity, po-sitive Likelihood ratio, NE-Gative likelihood ratio, diagnostic odds ratio and other indicators were used to determine the diagnostic efficacy of ultrasound and helical CT.

**Conclusion:**

This study is aimed at providing an evidence-based basis for clinicians to choose an appropriate or optimal diagnostic method by comparison of the accuracy and practicality between spiral CT and ultrasound in the diagnosis of intestinal obstruction.

**Ethics and dissemination:**

The private information from individuals will not be published. This systematic review also will not involve endangering participant rights. Ethical approval is not required. The results may be published in a peer-reviewed journal or disseminated in relevant conferences.

**OSF Registration number:**

DOI 10.17605/ OSF.IO / Q5RNS.

## 1 Introduction

Intestinal obstruction is defined that intestinal contents caused by any cause that cannot function properly and pass through the intestine smoothly. With high clinical morbidity and mortality, intestinal obstruction is a common cause of acute abdominal pain, which the beginning is acute, the progress is quick.^[1]^ Approximately 15% of admissions are caused by acute abdominal pain in the US, and about 20% of acute surgical treatment results from intestinal obstruction.^[2]^ Clinical manifestations include nausea, emesis, colicky abdominal pain, and cessation of passage of flatus and stool.^[3]^ If lacking effective and positive treatments, patients with severe pain will have serious symptoms such as intestinal ischemic and necrosis, even perforated and shock. All of these threaten the lives of patients.^[4]^ The key point of treating intestinal obstruction is performing effective diagnosis to make sure the lesion and pathogenesis in time. All of these measurements are important for providing an early treatment regimen.^[5]^ With no specificity and sensibility of the clinal manifestations, physical examination, and laboratory examination results, iconography is widely applied to diagnose and determine treatment methods.^[6]^

A flat abdominal radiograph is often used as the first imageological examination for diagnosing acute abdominal pain and intestinal obstruction. Still, it lacks accuracy and sensitivity, especially in diagnosing a closed-loop, ischemic, or strangulating obstruction.^[2]^ Studies have suggested that X-ray is no longer used to evaluate abdominal symptoms.^[7]^ In stark contrast, however, ultrasonography and spiral CT are more accurate in diagnosing intestinal obstruction and providing important differential diagnosis.^[8]^ Spiral CT has become the preferred imaging method in patients with known or suspected obstruction depending on its ability to provide an all-around perspective of the intestinal tract, vascular system, mesentery, omentum, retroperitoneum, and peritoneum.^[2]^However, with the in-depth study of iconography, CT also has many problems such as delayed diagnosis, high cost, and radiation.

In recent years, ultrasound is more and more widely used in the diagnosis of intestinal obstruction. Some studies suggested that ultrasound is more accurate than CT in the diagnosis of intestinal obstruction.^[9,10]^ But some other studies suggested that the accuracy, obstruction site, cause of obstruction, and the coincidence rate of obstruction type diagnosed by spiral CT.^[11,12]^Currently, there is no unified conclusion on the accuracy and practicability of the above methods in indiagnosing intestinal obstruction. This systematic evaluation aims to evaluate the accuracy and practicability of the 2 methods in diagnosing intestinal obstruction to provide an evidence-based basis for clinicians.

## 2 Methods

### 2.1 Protocol register

This systematic review protocol and meta-analysis have been drafted under the guidance of the preferred reporting items for systematic reviews and meta-analyses protocols (PRISMA-P).^[13]^ Moreover, it has been registered on the open science framework (OSF) on October 23, 2020 (registration number: DOI 10.17605/ OSF.IO / Q5RNS).

### 2.2 Ethics

Since this is a protocol with no patient recruitment and personal information collection, the approval of the ethics committee is not required.

### 2.3 Eligibility criteria

#### 2.3.1 Types of studies

We will collect case-control studies and cohort studies of comparing ultrasound and spiral CT in the diagnosis of intestinal obstruction, and the language will be limited to Chinese and English.

#### 2.3.2 Object of study

Patients diagnosed with intestinal obstruction by ultrasound or spiral CT and confirmed by pathological examination according to the gold standard, regardless of nationality, race, age, gender, course of the disease, etc.

#### 2.3.3 Types of tests

Ultrasound examination was performed in the observation group and a spiral CT examination was performed in the control group, with no limitation on the type of inspection equipment. All patients accepted the accuracy of the imageological examination and pathological examination.

#### 2.3.4 Outcome indicators

Sensitivity (SEN), specificity (SPE), po-sitive likelihood ratio (+LR), ne-gative likelihood ratio (-LR), diagnostic odds ratio (DOR), and confidence interval (CI) of intestinal obstruction by ultrasound or spiral CT.

### 2.4 Exclusion criteria

1. Republished paper;
2. The published literature is abstract or data is incomplete after contacting the authors;
3. Meeting abstracts, comments, abstracts, reviews, case reports, animal experiments, etc;
4. Studies not validated by the Gold Standard.

### 2.5 Search strategy

“Ultrasound,” “spiral CT,” and “intestinal obstruction” in Chinese were searched in the Chinese database, including CNKI, WAN FANG, VIP, CBMDISC. “Ultrasound”, “Spiral CT”, “Spiral Computed Tomography”, “bowel obstruction”, “Intestinal obstruction” in English were searched in English database, including PubMed, EMBASE, Web of Science and the Cochrane Library. From the establishment of the database to October 2020, all Chinese and English literature on the comparison of accuracy and practicability between ultrasound and spiral CT in the diagnosis of intestinal obstruction. Take PubMed as an example, the search strategy is shown in Table 1.

**Table 1:** Search strategy in PubMed database.

Number	Search terms
#1	ultrasound [Title/Abstract]
#2	Spiral CT [Title/Abstract]
#3	Spiral Computed Tomography [Title/Abstract]
#4	#2 OR #3 OR
#5	Intestinal Obstruction [MeSH]
#6	Obstruction, Intestinal [Title/Abstract]
#7	bowel obstruction [Title/Abstract]
#8	#5 OR #6 OR #7
#9	#1 AND #4 AND #8

### 2.6 Filtration and extraction of data

**Figure 1:**
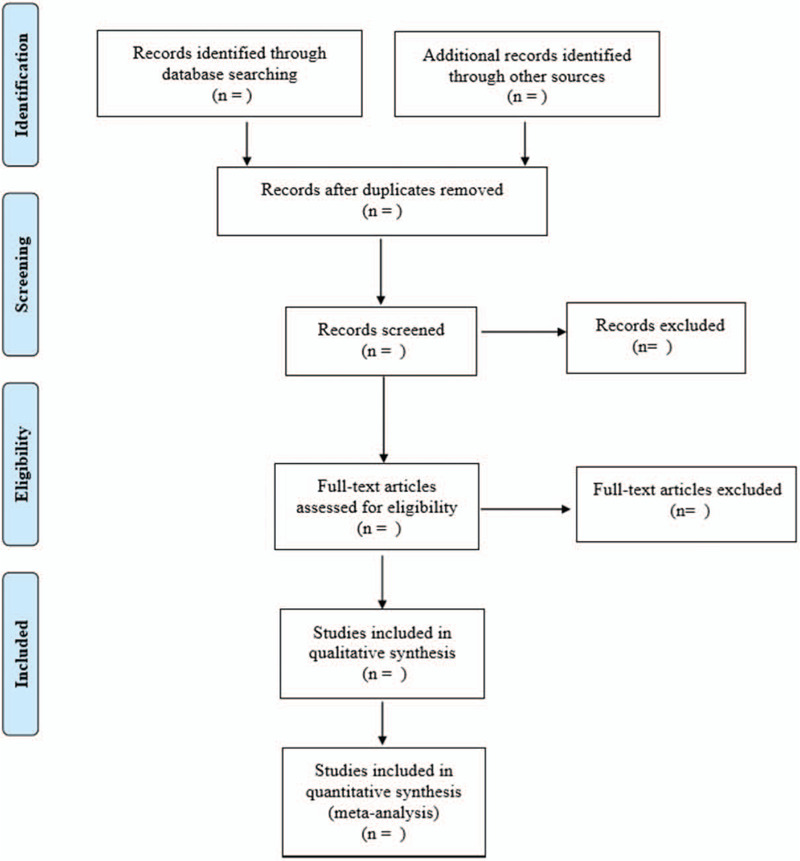
Flow diagram.

Referring to the method of research selection in version 5.0 of the Cochrane collaboration Network system Evaluator Manual, according to the PRISMA flow chart, the 2 researchers used the EndNote X9 document management software to independently screen and check the literature according to the above inclusion and exclusion criteria, and check each other, if there were different opinions, negotiate with a third party to resolve the differences. According to the inclusion and exclusion criteria in literature, relevant data were extracted independently from all eligible studies and recorded in Excel 2013. ①The basic features of the included studies including the first author, published year, language, country, case number, imaging method, the gold standard, and so on.②Key elements of bias risk assessment.③The concerned outcome measurement data, such as true positive values, false-positive values, false-negative values, etc. The screening process of the literature is shown in Figure 1.

### 2.7 Quality evaluation of literature

Evaluated the bias risk of the included literature by QUADAS-2 evaluation standard of quality.^[14]^ The tool consists of bias evaluation and applicability, including case selection, tests remained to be evaluated, gold standard, the process of cases, and progress. “high risk,” “low risk,” or “unclear” was given for all areas covered. Evaluation of risk bias according to the performance of the included literature in the above evaluation items and cross-checked after completing respectively. A discussion was required if there was a difference. If no agreement can be reached, a third party researcher was required.

### 2.8 Statistical analysis

#### 2.8.1 Data analysis and processing

RevMan 5.3 and Meta Disc 1.4 was used in meta-analysis. *I*^2^ index was used to judge the size of heterogeneity. If *P* > .10 and *I*^2^ < 50% indicated the heterogeneity is small among studies, then the fixed-effect model was used for the merger. If *P* ≤ .10 and *I*^2^ > 50% indicated the heterogeneity is large among studies, then random-effects model was used for the merger. Calculated and merged sensitivity (SEN), specificity (SPE), positive likelihood ratio (+LR), negative likelihood ratio (-LR), diagnostic odds ratio (DOR) and confidence interval (CI). Drew and merged the summary receiver operating characteristic curve (SROC), then got area under the curve (AUC). Meanwhile, sensitivity analysis was performed to evaluate the results stability by excluding the included references one by one.

#### 2.8.2 Dealing with missing data

If there is a lack of data in the article, please contact the author by email to supplement relevant information. If there is no way to contact authors or get relevant data, a descriptive analysis will be conducted instead of meta-analysis.

#### 2.8.3 Subgroup analysis

This study will carry out a subgroup analysis based on the different patient characteristics, index and reference tests, and outcome indicators.

#### 2.8.4 Assessment of publication bias

If there is more than 10 studies, the Deek funnel plot will be conducted to evaluate the potential degree of publication bias.^[15]^ Moreover, Egger test and Begg test were used for the evaluation of potential publication bias.

#### 2.8.5 Grading the quality of evidence

Grading of Recommendation Assessment, Development, and Evaluation (GRADE) will be conducted to grade the evidence for outcome measures.^[16]^ The evaluation content includes bias risk, indirectness, inconsistencies, uncertainty, and publication bias. The quality of evidence will be rated as high, medium, low, or very low.

## 3 Discussions

Intestinal obstruction is a common type of acute abdomen, mainly caused by intestinal contents that cannot passing through smoothly, which leads to pathological changes. Therefore, intervention treatment is needed in time. A clear diagnosis is important because different intestinal obstruction causes, pathogenic site, and degrees should be conducted with different therapeutic schedules.

During the examination of intestinal obstruction by ultrasound, the continuous and obvious dilatation of intestinal canal, thickened edema of the intestinal wall, hydrops and pneumatosis in the enteric cavity and enhancement or weak even disappearance of bowel peristalsis were observed. The blood supply changes of mesenteric vessels and intestinal wall and ascites characteristics were also observed.^[4]^The characteristic ultrasonic images of intestinal obstruction include “gas-liquid stratification”, “images like a fishbone”, “images like keyboard”, “images like bread”, “images like a concentric circle”, “images like sleeve sign”, etc.^[17,18]^The biggest limitations on ultrasound are obesity and the presence of large amounts of intestinal gas.^[19,20]^ Some studies pointed out the difference between the 2 methods is that CT can clearly show the specific parts of the intestinal obstruction and have a high value on the judgment of localization and causes of the lesion site. Still, ultrasound can show the specific structure of the intestinal wall in the abdominal cavity by the acoustic window to clearly observe intestinal obstruction.^[12,21]^

There are advantages and disadvantages in diagnosing intestinal obstruction by Ultrasound and spiral CT, which are controversial in the clinical choice. And there is no systematic review of the subject currently. This study will systematically and comprehensively evaluate the accuracy and practicability of ultrasound and spiral CT in intestinal obstruction diagnosis based on the summary of current studies. The results of this study will provide an evidence-based basis for judging the accuracy and practicality of ultrasound and spiral CT in the diagnosis of intestinal obstruction.

However, this systematic review has some limitations. Differences in the frequency and thickness of spiral CT scanning of the ultrasound probes used in the included studies may result in certain clinical heterogeneity. Due to language competence limitations, we can only search for English and Chinese literature and may ignore studies or reports in other languages.

## Author contributions

**Data curation:** Qingya Yang, Fan Zhao.

**Funding acquisition:** Fan Zhao.

**Investigation:** Qingya Yang.

**Project administration:** Long Su.

**Resources:** Qingya Yang.

**Software:** Junfeng Qi.

**Writing – original draft:** Qingya Yang.

**Writing – review & editing:** Qingya Yang, Fan Zhao.

## Abbreviations

Abbreviations: +LR = po-sitive likelihood ratio, AUC = area under the curve, CBMDISC = China Biology Medicine disc, CI = confidence interval, CMB = Chinese Biological and Medical database, CNKI = China National Knowledge Infrastructure, CT = computerized tomography, DOR = diagnostic odds ratio, -LR = ne-gative likelihood ratio, NOS = Newcastle-Ottawa Quality Assessment Scale, OSF = open science framework, PRISMA-P = Preferred Reporting Items for Systematic Review and Meta-Analysis Protocols, QUADAS = Quality Assessment of Diagnostic Accuracy Studies, RCTs = randomized controlled trials, RR = relative risk, SEN = sensitivity, SPE = specificity, SROC = summary receiver operating characteristic curve, WMD = weighted mean difference.
